# Metagenomic mining pectinolytic microbes and enzymes from an apple pomace-adapted compost microbial community

**DOI:** 10.1186/s13068-017-0885-y

**Published:** 2017-08-22

**Authors:** Man Zhou, Peng Guo, Tao Wang, Lina Gao, Huijun Yin, Cheng Cai, Jie Gu, Xin Lü

**Affiliations:** 1 College of Food Science and Engineering, Northwest A&F University, Yangling, Shaanxi Province China; 2 College of Information Engineering, Northwest A&F University, Yangling, Shaanxi Province China; 3 College of Natural Resources and Environment, Northwest A&F University, Yangling, Shaanxi Province China

**Keywords:** Lignocellulosic biofuel, Pectin, Metagenomic, Pectinolytic microbes and enzymes, Compost habitat

## Abstract

**Background:**

Degradation of pectin in lignocellulosic materials is one of the key steps for biofuel production. Biological hydrolysis of pectin, i.e., degradation by pectinolytic microbes and enzymes, is an attractive paradigm because of its obvious advantages, such as environmentally friendly procedures, low in energy demand for lignin removal, and the possibility to be integrated in consolidated process. In this study, a metagenomics sequence-guided strategy coupled with enrichment culture technique was used to facilitate targeted discovery of pectinolytic microbes and enzymes. An apple pomace-adapted compost (APAC) habitat was constructed to boost the enrichment of pectinolytic microorganisms.

**Results:**

Analyses of 16S rDNA high-throughput sequencing revealed that microbial communities changed dramatically during composting with some bacterial populations being greatly enriched. Metagenomics data showed that apple pomace-adapted compost microbial community (APACMC) was dominated by *Proteobacteria* and *Bacteroidetes*. Functional analysis and carbohydrate-active enzyme profiles confirmed that APACMC had been successfully enriched for the targeted functions. Among the 1756 putative genes encoding pectinolytic enzymes, 129 were predicted as novel (with an identity <30% to any CAZy database entry) and only 1.92% were more than 75% identical with proteins in NCBI environmental database, demonstrating that they have not been observed in previous metagenome projects. Phylogenetic analysis showed that APACMC harbored a broad range of pectinolytic bacteria and many of them were previously unrecognized.

**Conclusions:**

The immensely diverse pectinolytic microbes and enzymes found in our study will expand the arsenal of proficient degraders and enzymes for lignocellulosic biofuel production. Our study provides a powerful approach for targeted mining microbes and enzymes in numerous industries.

**Electronic supplementary material:**

The online version of this article (doi:10.1186/s13068-017-0885-y) contains supplementary material, which is available to authorized users.

## Background

High worldwide demand for energy and increasing concerns over global climate change have prompted the development of sustainable and environmentally friendly energy [16, 45]. Lignocellulosic biofuel, which derived from the most abundant renewable organic material on our planet, represents a promising alternative to fossil fuels [12, 15]. However, the major obstacles to industrial-scale production of biofuel from lignocellulosic feedstocks lie in the recalcitrant nature of biomass toward enzymatic breakdown and the relatively low activity of currently available hydrolytic enzymes [15, 44].

Pectin is one of the plant cell wall components. It is abundant in the middle lamella and primary cell walls, though presents at low levels in secondary walls [8]. For the cell walls of pectin-rich biomass, for example apple pomace, it contains 12–35% pectin on a dry weight basis [11]. In plant biomass, pectin embeds in the cellulose–hemicellulose network of the cell wall and regulates intercellular adhesion like glues [16]. It is the complex matrix of pectin that masks cellulose and/or hemicellulose through hydrogen bonding interactions [53], and blocks their accessibility to degradative enzymes [8], thus resulting in plant biomass that is less susceptible to degradation and more recalcitrant to deconstruction [23]. As a result, degradation of pectin in lignocellulosic materials has been established as essential for efficient bioconversion of lignocellulose [47]. Recently, the reduction of bulk percentage of pectin through genetic manipulation or enzymatic means has been proved to reduce the recalcitrance and accelerate the lignocellulose saccharification of herbaceous plants [23], *Arabidopsis* [12], switchgrass [8], and woody biomass [3, 4].

Removal of pectin can be achieved in physical, chemical, or biological manner. Biological hydrolysis of pectin by pectinolytic microbes and enzymes is favored as it is environmentally benign and energy efficient [26]. Pectinolytic enzymes have multiple benefits in the efficient hydrolysis of lignocellulosic materials: first, yield of fermentable sugars by hydrolysis of pectin itself [4]; second, facilitation of sugar release by disrupting the pectin network around cellulose and lignin [23], and exposure of other polymers to degradation by hemicellulases and cellulases [30]; third, improvement of cell wall porosity [3] and reduction of mechanical strength because of its crosslinking and water complexation features [47]. Especially for pectin-rich lignocellulosic biomass which also could serve as the feedstock for lignocellulosic biofuel [30], for instance apple pomace, pectinolytic enzymes will play a more prominent role.

Despite pectinolytic enzymes playing a crucial part in the lignocellulosic biofuel production, most of the currently available pectinolytic enzymes are costly, inefficient, and susceptive to fluctuations in feedstock [5]. In consequence, search for microbes and enzymes from naturally evolved pectinolytic microbial communities offers a promising strategy for the discovery of new pectinolytic enzymes. Given the unique features of compost habitat, there is tremendous potential to discover robust organisms and novel enzymes which tolerate harsh pretreatment scenarios under industrial conditions [2]. Thus, compost is considered as one of the most attractive DNA pools for target gene discovery [2, 24].

Metagenomics, which directly analyzes the total DNA from environmental samples, provides a powerful strategy in unveiling the novel microbes and enzymes in microbial communities without the technical challenges of cultivation [15, 37]. However, as environmental samples generally hold a huge reservoir of extensive microbes and enzymes, it is unfeasible to characterize them accurately. Hence, to reduce the complexity of metagenomic datasets, render further assembly more amenable, and more importantly, improve the specificity of the sample’s DNA, the oriented enrichment culture technique is essential to be employed for the establishment of microbial consortia with desired functionality [28]. In this manner, the enzyme repertoire of enriched consortia can be tailored to degrade specific feedstock [52]. Since apple pomace is an pectin-rich lignocellulosic biomass [30], pectinolytic enzymes could be exploited from the established pectinolytic microbes which are selectively enriched in abundance from compost communities by cultivation with apple pomace as the sole carbon source.

**Fig. 1:**
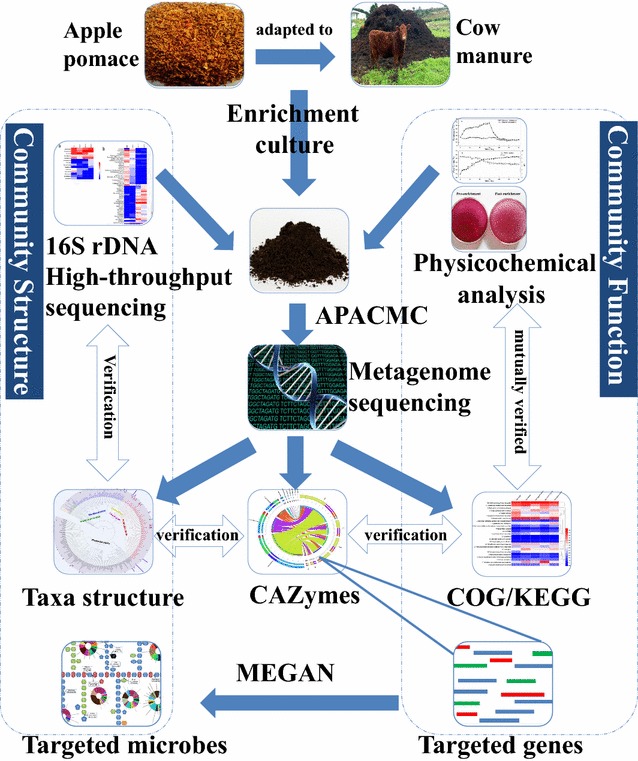
A pipeline of metagenomics sequence-guided strategy coupled with enrichment culture technique used in this study

In this study, a metagenomic sequence-guided strategy combined with enrichment culture technique was used to targetedly discover the pectinolytic microbes and enzymes from an apple pomace-adapted compost microbial community (APACMC). The pipeline of this strategy is shown in Fig. 1. Firstly, the unique APACMC was constructed from the cow manure compost habitat to boost the enrichment of pectinolytic microorganisms. The dynamic microbial changes of APACMC were characterized by 16S rDNA high-throughput sequencing. Secondly, a targeted metagenomic approach was applied to facilitate the identification of pectinolytic microbes and enzymes. A more accurate microbial taxonomic analysis and function characterization were conducted. Thirdly, the metagenome sequences were annotated and phylogenetically affiliated against carbohydrate-active enzymes (CAZymes) database. Finally, after the specific investigation of genes related to pectinolytic CAZymes and their taxonomic affiliations, the robust microorganisms and novel enzymes processing the degradation of pectin were identified.

## Results and discussion

### Changes in physicochemical properties during composting

**Fig. 2:**
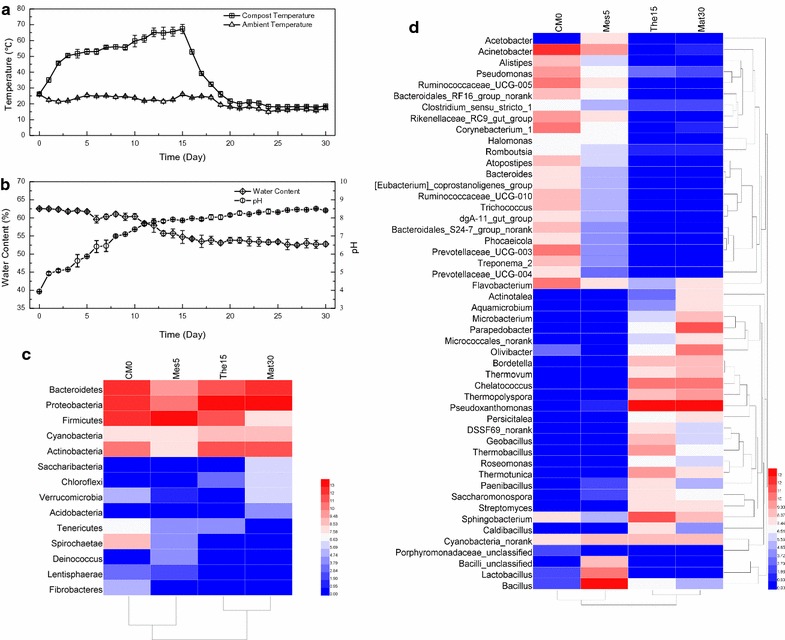
The changes of physicochemical properties and bacterial communities during composting (**a** temperature; **b** water content and pH; **c** at the phylum level; **d** at the genus level)

The variations of physicochemical properties during composting are strongly associated with the biological reactions involving organic matter, and thus, these changes reflect the microbial activity and progress of the composting process [41]. The dynamic changes in physicochemical properties (i.e., temperature, water content, and pH) during the apple pomace-adapted compost (APAC) process are illustrated in Fig. 2. During the 30-day enrichment period (Fig. 2a), the temperature of the APAC pile maintained at 25–35 °C for 24 h to allow the compost microbes to establish, then it rapidly reached 60–70 °C to trigger the thermophilic phase. After the temperature reached 68 °C at day 15, the temperature declined gradually back to the ambient temperature over the rest 15 days to trigger the cooling and maturation phase. As shown in Fig. 2b, the initial pH of APAC was in the range of 3.8–4.0 as the acid–base nature of apple pomace. Eventually, the pH value of APAC gradually rose to approximately 8.5. The escalating pH during composting may be attributed to the release of ammonia, methanol, and the decomposition of organic acids of apple pomace [51]. The water content of APAC dropped fast at the early stages and then declined slowly. The small variations of pH and water content at the end of enrichment indicated that the microorganisms were still active and the degradation of apple pomace continued.

### Changes in bacterial community structure during composting

To characterize the changes of microbiota structure during composting, 16S rDNA sequencing on the representative samples of different phases, i.e., CM0 (day 0), Mes5 (day 5 of Mesophilic), The15 (day 15 of Thermophilic), and Mat30 (day 30 of Maturation), was performed. As expected, the sample CM0 had the highest α diversity (OUT numbers and Chao1 estimator) while The15 had the lowest (Additional file 1: Table S1). Although the main phyla throughout the entire composting process did not change greatly (Fig. 2c), namely *Actinobacteria*, *Bacteroidetes*, *Proteobacteria, Firmicutes*, and *Cyanobacteria*, the main genera varied dramatically with some bacterial populations being greatly enriched (Fig. 2d). At the genus level, the bacterial community profiles of the main genera were clustered into two groups, which the bacterial community structures in The15 and Mat30 differed remarkably from CM0. The genera *Acinetobacter* (21.9%), *Planococcaceae unclassified* (8.0%), and *Ruminococcaceae* UCG-005 (6.9%) were the dominant in the CM0, whereas they declined to a very low level or completely disappeared after day 15 (Fig. 2d). By contrast, the genera *Pseudoxanthomonas* (36.7%), *Parapedobacter* (16.8%), *Chelatococcus* (7.2%), *Olivibacter* (4.7%), and *Sphingobacterium* (2.4%) were enriched in the composting process. The evolution of specific populations reflected that APAC had adapted to apple pomace degradation. Furthermore, most of the species abundant in Mat30 have been detected showing highly positive correlations on lignocellulose/pectin-degrading activities [9, 36, 44, 52]. The predominance of lignocellulolytic or pectinolytic species suggests that APACMC has potential to degrade lignocellulose and pectin effectively.

Moreover, the pectinolytic activities of APAC increased dramatically after the composting process, which preliminarily proved the effectiveness of the microbial enrichment (Additional file 2: Fig. S1). Further, it was showed that 83.25% pectin was degraded through 30-day composting. Consequently, APACMC was supposed to be successfully established with pectinolytic capability by means of the enrichment culture technique we adopted.

### Microbial diversity in APACMC metagenome

To obtain more detailed information on the diversities of pectinolytic microbes and genes encoding pectinolytic enzymes in APACMC, shotgun sequence of Mat30 was performed by using Illumina HiSeq4000 platform. Metagenomic sequencing of APACMC yielded 89,623,103 reads after quality filtering. After assembly, 272,516 predicted ORFs with the average length of 668 bp were obtained (Additional file 3: Table S2). The analysis of metagenomic datasets showed that APACMC was predominately composed of bacterial members (~99.7%), along with very few archaea, eukarya, and uncharacterized organisms. This could be explained by the fact that fungal activities were precluded as APACMC sustained high temperatures between 55 and 68 °C (Fig. 2a).

**Fig. 3:**
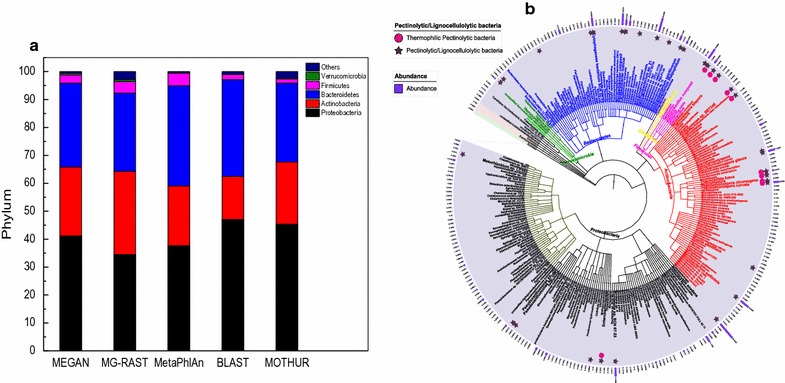
Taxonomy composition (**a**) and phylogenetic tree (**b**) of APACMC metagenome

To estimate the microbial diversity more accurately, various taxonomic protocols such as MEGAN, MetaPhlAn, and MG-RAST were used, while minor differences in the rank abundance order were observed. Taxonomic analysis revealed that APACMC was primarily consisted of members from phyla *Proteobacteria*, *Bacteroidetes*, *Actinobacteria*, and *Firmicutes* (Fig. 3a), which agreed with the result of 16S rDNA sequencing (Mat30 in Fig. 2c). Several previous studies have reported that thermophilic compost communities contain high-abundance genera within these phyla [18, 36]. Other phyla, such as *Verrucomicrobia*, *Cyanobacteria*, and *Planctomycetes* were presented at very low abundances, which together accounted for only 1.04% of the total sequences. Meanwhile, around 623 predicted genes could not be assigned to a definite bacteria phylum, which may belong to yet uncharacterized bacteria.

The prevalence of genus *Sphingobacterium* (17.11%), *Pseudoxanthomonas* (13.46%), *Bordetella* (4.83%), *Microbacterium* (2.23%), *Pedobacter* (2.13%), *Niabella* (2.10%), and *Thermobacillus* (1.33%) was in accordance with previous studies, which have found these species to be major components of bacterial consortia with high cellulolytic or pectinolytic activity in compost habitats [9, 18, 36, 40]. The taxonomic compositions at genus level were also consistent with the result of 16S rDNA sequencing (Mat30 in Fig. 2d). In addition, it was found some *Enterobacteriaceae* species also had high capacity for metabolizing pectin, such as genera *Yersinia*, *Klebsiella*, *Dickeya*, and *Pectobacterium* [1]. Moreover, the thermophilic pectinolytic bacteria, such as *Actinomadura* [38], *Bacillus* [25], *Geobacillus* [43], *Streptomyces* [50], *Thermomonospora*, and *Thermobifida* [44] were also detected.

To gain further insight into the diversity of APACMC, the metagenomic dataset was taxonomically profiled at the species level (Fig. 3b). The result showed that *Nannocystis exedens* (30.44%, marked with the highest bar, see Fig. 3b) was the most prevalent species accompanying with *Sphingobacterium* sp. 21 (23.33%), *Parapedobacter composti* (18.97%), *Cytophagaceae bacterium SCN* 52-12 (13.69%), *Thermobacillus composti* (18.24%) and *Saccharomonospora glauca* (11.91%). It was reported that most of them (marked with asterisk, see Fig. 3b) are proficient degraders of lignocellulose [18, 36, 40]. Simultaneously, a number of thermophilic pectinolytic bacteria (marked with solid circle, see Fig. 3b) including *Thermobispora bispora* [44], *Thermomonospora curvata* [50], and *Thermobifida fusca* [52] were found. Collectively, large proportion of lignocellulolytic and pectinolytic microorganisms further confirms that APACMC has possessed the targeted functions.

### Functional profiles of predicated genes in APACMC metagenome

**Fig. 4:**
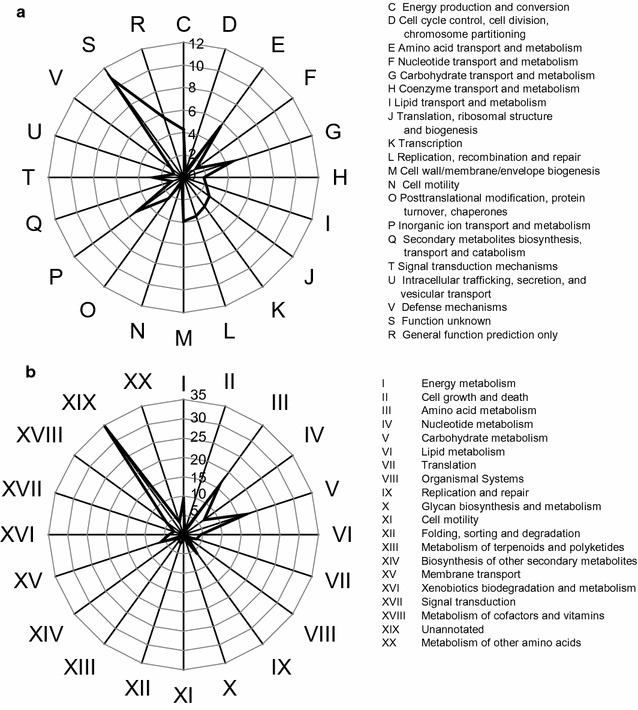
COG (**a**) and KEGG (**b**) functional categories of APACMC metagenome

Annotations by the MG-RAST pipeline revealed that 99.98% of the predicted genes were protein coding, among which 81.01% had been assigned a putative function. The COG and KEGG repertoire of the predicted genes was analyzed to assess the primary functions of these genes in APACMC. The COG categories analysis (Fig. 4a) showed that APACMC was enriched for amino acid metabolism (8.8% in all COG functional categories), general function (8.7%), inorganic ion metabolism (7.9%), carbohydrate transport and metabolism (7.1%), energy production and conversion (6.6%), and cell wall/membrane/envelope biogenesis (6.1%). The comparative COG analysis of the APACMC with another four well-known lignocellulose-degrading consortia from rain forest compost, switchgrass-adapted compost [2], Sao Paulo zoo park compost [24], and rice straw-adapted compost [36] revealed that they shared similar metabolic patterns, particularly associated with carbohydrates and amino acids transport and metabolism (Additional file 4: Fig. S2). The KEGG ontology exhibited analogous patterns (Fig. 4b), where carbohydrate metabolism (17.0%), amino acid metabolism (16.0%), energy metabolism (9.4%), nucleotide metabolism (6.6%), and membrane transport (6.2%) were abundant. Generally, these observations indicate that APACMC has successfully enriched several desired functional capacities, especially, for carbohydrate metabolism.

In order to get more detailed information about the decomposition of pectin, specific COGs involved in pectin transport and metabolism were further analyzed (Additional file 5: Table S3). APACMC harbored a broad spectrum of genes involved in the metabolism of different monosaccharide building blocks of pectin (e.g., arabinose, fucose, galactose, mannose, rhamnose, xylose, etc.), all of which accounted for 25.8% of the COG subcategory G (Carbohydrate transport and metabolism). Additionally, the genes associated with carbohydrate transporters and phosphotransferase systems were also very plentiful. For example, ABC-type sugar transport system, permease, TonB, and phosphotransferase system, which are responsible for the uptake, transport, and phosphorylation of sugars [18, 44], took up 7.2, 10.6, 2.6, and 1.0% of the COG subcategory G, respectively. In summary, the rich diversity of gene functions in carbohydrate transport and metabolism indicates that APACMC has enriched a great potential for the degradation of pectin.

### The diversity, abundance, and phylogenetic distribution of CAZymes in APACMC metagenome

It is well established that the plant biomass-degrading capacities of microbial consortia are closely related to genes encoding CAZymes [52]. To gain an overview of microbial degradation of main polymers in apple pomace, we screened APACMC metagenome for the discovery of microorganisms and genes encoding CAZymes. All the candidate genes of APACMC metagenome were searched against the CAZy database using dbCAN [49] for the presence of at least one relevant catalytic domain or carbohydrate-binding module, rather than overall sequence similarity to known CAZymes. The results showed that APACMC harbored a total of 9274 different CAZyme genes, which distributed heterogeneously among glycoside hydrolases (GHs, 35.6%), glycosyltransferases (GTs, 26.9%), carbohydrate esterases (CEs, 17.5%), carbohydrate-binding modules (CBMs, 13.3%), auxiliary activities (AAs, 4.6%), and polysaccharide lyases (PLs, 2.1%) (Additional file 6: Table S4).

**Fig. 5:**
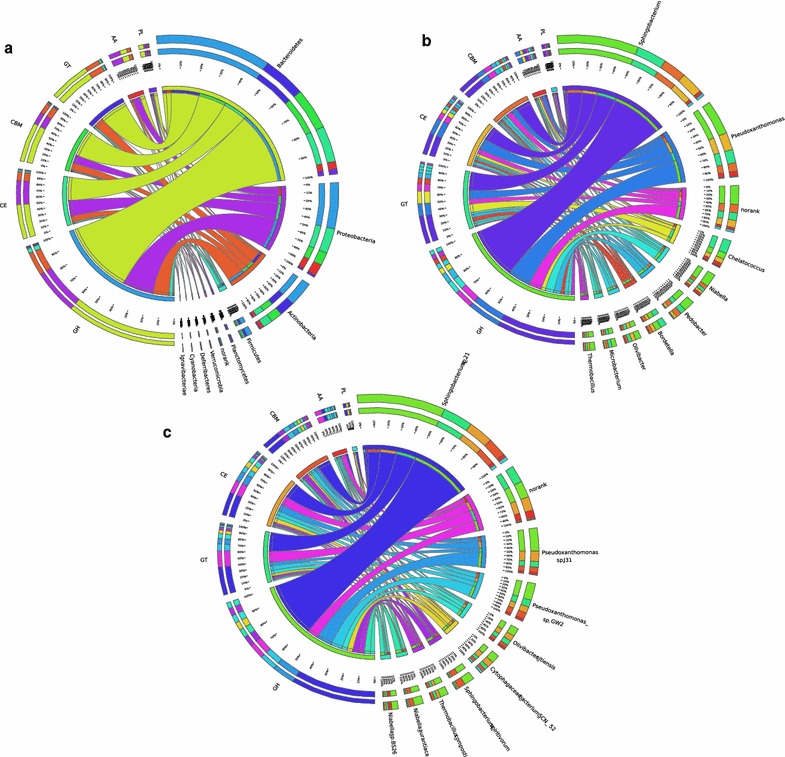
Phylogenetic distributions of CAZymes in the most abundant members in APACMC (**a** at phylum level; **b** at genus level; **c** at species level)

To link metabolic functions to abundant consortia members, all the CAZyme genes of APACMC were taxonomically classified. The phylogenetic distribution of CAZyme genes showed that the abundance of CAZyme genes varied across bacterial phyla and most of them were derived from *Bacteroidetes* (51.86%), *Proteobacteria* (30.82%), *Actinobacteria* (13.52%), and *Firmicutes* (2.31%) (Fig. 5a). Notably, members of *Bacteroidetes* were predominant in the CAZymes classes (GHs, GTs, CEs, PLs, and CBMs) of APACMC, which was remarkably different from those of rice straw-adapted compost (*Actinobacteria*) [44] and corn stover-adapted compost (*Proteobacteria*) [52].

As the CAZyme genes were unevenly distributed within each phylum, the extensive phylogenetic distributions of CAZyme genes at lower taxonomic levels were further investigated. In addition to *Sphingobacterium* and *Niabella* of the phylum *Bacteroidetes*, CAZyme genes were also abundant in *Pseudoxanthomonas* and *Chelatococcus* of the phylum *Proteobacteria*, *Microbacterium* of the phylum *Actinobacteria*, as well as *Thermobacillus* of the phylum *Firmicutes* (Fig. 5b). At the species level, six different species of phylum *Bacteroidetes*, which accounted for 25.06% of the total CAZyme genes, were present in the top-10 richest members harboring CAZyme genes (Fig. 5c). This finding indicates that members of *Bacteroidetes* possess a much abundant and wider range of CAZyme catalog in APACMC. Besides, the CAZyme genes were also detected in uncharacterized species of *Chelatococcus* (5.04%), *Pseudoxanthomonas* sp. GW2 (3.62%), and *Pseudoxanthomonas* sp. J31 (4.48%) of the phylum *Proteobacteria* and *Thermobacillus composti* (1.57%) of the phylum of *Firmicutes*.

The phylogenetic distributions of CAZyme genes corresponded well to the structure of the ecologically dominant species in APACMC, which confirms the assumption that the functional traits of consortia have a direct correlation with their taxonomic profiles [44]. In conclusion, the CAZymes profile reveals that polysaccharides of apple pomace are decomposed by the predominant *Bacteroidetes* in cooperation with *Proteobacteria*, *Actinobacteria*, and *Firmicutes*. Together with the COG profiles for glycan degradation, the diverse repertoire of CAZymes provides a basis for a collaborative system tailored to the processing and metabolizing of apple pomace in the compost habitat.

### Mining for pectinolytic enzymes

**Fig. 6:**
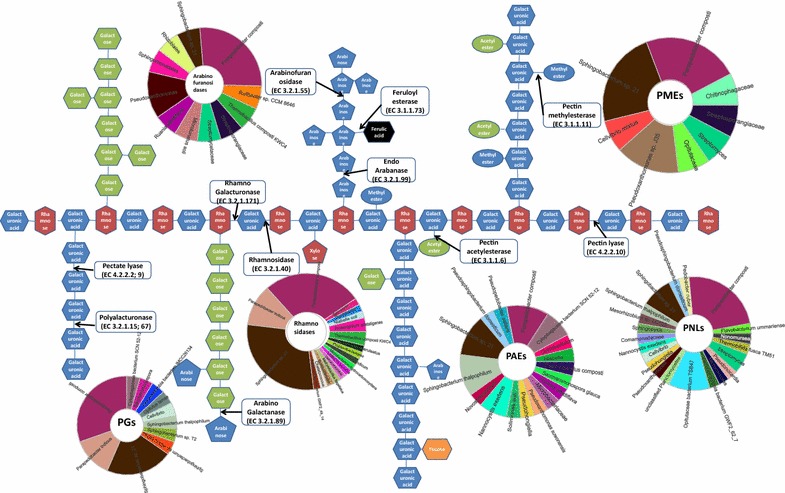
Schematic representation of pectin structure (Schematic representation of pectin structure was modified from [19]) and the phylogenetic affiliation (The phylogenetic affiliations of key pectinolytic enzymes in APACMC were visualized by MEGAN6) of key pectinolytic enzymes in APACMC

Pectin is the major composition of apple pomace and is an extremely structurally complex polysaccharide, which is constituted of as many as 17 different monosaccharides and more than 20 different linkages [5]. The representative structure of pectin is schematically shown in Fig. 6. It is basically composed of homogalacturonan (HG), rhamnogalacturonan I (RG-I), the substituted galacturonans rhamnogalacturonan II (RG-II), and xylogalacturonan (XGA) [19]. Due to its complex and heterogeneous structure, the efficient and complete degradation of pectin involves a battery of enzymes which act specifically and synergistically. These pectin-degrading enzymes are classified as de-polymerases (hydrolases and lyases), pectinesterases, and de-branching enzymes based on the action mode and site.

The different types of pectinolytic enzymes and their cleavage sites are depicted in Fig. 6. The degradation of pectin is caused by the de-esterification of methoxyl groups, affecting the texture and rigidity of the cell wall [1]. Pectin methylesterases (PMEs, EC 3.1.1.11) remove the methyl groups from the HG backbone to give access to de-polymerases, while pectin acetylesterases (PAEs, EC 3.1.1.6) remove acetyl groups from acetylated HG and RG [32]. Hydrolases (polygalacturonases PGs, EC 3.2.1.15, 67 and 82) and lyases (pectin lyases: PNLs, EC 4.2.2.10 and pectate lyases: PELs, EC 4.2.2.2 and 9) preferentially degrade the α-1,4-glycosidic bonds of HG/XGA backbones by hydrolysis and β-elimination, respectively. Similarly, in the initial deconstruction of pectin, PNLs also play an essential role since it is the only enzyme that can cleave the α-1, 4 bonds of highly esterified pectin without prior actions of other enzymes. De-branching enzymes are responsible for the cleavage of the backbone or lateral chains of RG-I and RG-II [5]. Rhamnogalacturonases (EC 3.2.1.171, 173 and 174) and rhamnogalacturonan lyases (RGLs: EC 4.2.2.23 and 24) are involved in the cleavage of the RG-I backbone; α-l-rhamnosidases (EC 3.2.1.40) act on hydrolytic cleavage of the RG chain at non-reducing end releasing rhamnose; arabinofuranosidases (EC 3.2.1.55) attack on α-l-arabinofuranosides, α-l-arabinans, arabinoxylans, and arabinogalactans; arabinogalactanases (EC 3.2.1.89) and β-galactosidases (EC 3.2.1.23) act randomly on the galactan core of AGs; and feruloyl esterases (EC 3.2.1.73) are needed to release the ferulic acid attached to C-2 of arabinose and C-6 of galactose.

According to the results of CAZymes annotation, we found an extremely abundant of genes associated with the complete degradation of pectin (Additional file 7: Table S5). A total of 1756 entries were identified as encoding pectinolytic enzymes, which took up to 18.93% of the total CAZy genes. As summarized in Table 1, these entries contained 105 PLs candidates from 6 families, 881 GHs mainly from 17 families, 537 CEs from 5 families, and 233 CBMs from 5 families. Compared to another two compost habitats (Table 1), the catalog of pectinolytic enzymes in APACMC was much more abundant and diverse than RSA (843 candidates from 31 families) and EMSD5 (398 candidates from 28 families), indicating that APACMC has a better potential for pectin degradation based on CAZyme inventory. As shown in Table 1, a large panel of pectinolytic enzymes was found, such as PGs, PNLs, PELs, RGLs, PMEs, PAEs, α-l-rhamnosidases, arabinofuranosidases, arabinogalactanases, and β-galactosidases. Furthermore, we also detected a wealth of CBMs which possibly associate to pectin degradation. For example, some members of family CBM32 have been found to bind oligogalacturonides to counteract the loss of binding affinity between thermophilic pectinases and their substrates at elevated temperature [1, 52]. These findings indicate that APACMC exhibits a collaborative enzymatic system efficient in the complete degradation of pectin.

**Table 1:** Summary of pectinolytic enzymes in three metagenomes

Pectinolytic enzymes	CAZy family		Predominant activity	This study	RSA^a^	EMSD5^b^
Depolymerizing enzymes	Glycoside hydrolases (GHs)	GH4	Exo-polygalacturonase (EC 3.2.1.67)	14	21	24
		GH28	Polygalacturonase (EC 3.2.1.15); exo-polygalacturonase (EC 3.2.1.67); exo-polygalacturonosidase (EC 3.2.1.82); rhamnogalacturonase (EC 3.2.1.171); rhamnogalacturonan α-1,2-galacturonohydrolase (EC 3.2.1.173); rhamnogalacturonan α-l-rhamnopyranohydrolase (EC 3.2.1.174)	32	11	3
	Polysaccharide lyases (PLs)	PL1	Pectate lyase (EC 4.2.2.2); exo-pectate lyase (EC 4.2.2.9); pectin lyase (EC 4.2.2.10)	30	8	4
		PL3	Pectate lyase (EC 4.2.2.2)	2	4	0
		PL4	Rhamnogalacturonan lyase (EC 4.2.2.-)	0	0	1
		PL9	Pectate lyase (EC 4.2.2.2); exopolygalacturonate lyase (EC 4.2.2.9)	12	15	4
		PL10	pectate lyase (EC 4.2.2.2)	13	8	0
		PL11	Rhamnogalacturonan endolyase (EC 4.2.2.23); rhamnogalacturonan exolyase (EC 4.2.2.24)	18	10	1
		PL22	Oligogalacturonate lyase/oligogalacturonide lyase (EC 4.2.2.6, 9)	30	21	12
Pectinesterases	Carbohydrate esterases (CEs)	CE1	Feruloyl esterase (EC 3.1.1.73)	482	223	82
		CE8	Pectin methylesterase (EC 3.1.1.11)	17	8	6
		CE12	Pectin acetylesterase (EC 3.1.1.6); rhamnogalacturonan acetylesterase (EC 3.1.1.86)	34	4	8
		CE13	Pectin acetylesterase (EC 3.1.1.6)	2	0	0
		CE16	Pectin acetylesterase (EC 3.1.1.6)	4	0	0
De-branching enzymes	Glycoside hydrolases (GHs)	GH1	β-Glucosidase (EC 3.2.1.23)	60	63	44
		GH2	β-Glucosidase (EC 3.2.1.23); α-L-arabinofuranosidase (EC 3.2.1.55)	76	27	19
		GH3	β-Glucosidase (EC 3.2.1.23); α-l-arabinofuranosidase (EC 3.2.1.55)	143	83	32
		GH10	Endo-1,4-β-xylanase (EC 3.2.1.8); endo-1,3-β-xylanase (EC 3.2.1.32)	50	37	6
		GH35	β-Glucosidase (EC 3.2.1.23)	9	7	1
		GH42	β-Glucosidase (EC 3.2.1.23)	33	14	9
		GH43	α-l-Arabinofuranosidase (EC 3.2.1.55); arabinanase (EC 3.2.1.99)	205	51	33
		GH51	α-l-Arabinofuranosidase (EC 3.2.1.55)	40	24	4
		GH53	Arabinogalactanase (EC 3.2.1.89)	8	6	6
		GH59	β-Glucosidases (EC 3.2.1.23)	2	2	0
		GH62	α-l-Arabinofuranosidase (EC 3.2.1.55)	2	6	0
		GH78	α-l-Rhamnosidase (EC 3.2.1.40)	92	39	8
		GH105	Unsaturated rhamnogalacturonyl hydrolase (EC 3.2.1.172)	35	4	5
		GH106	α-l-Rhamnosidase (EC 3.2.1.40)	30	6	3
		GH127	β-l-Arabinofuranosidase (EC 3.2.1.185)	51	17	11
	Carbohydrate-binding modules (CBMs)	CBM13	Arabinanase (GH43D;RUM_09280); feruloyl esterase I (FaeI;CGSCsYakCAS_18248); pectate lyase B (PelB)	25	14	24
		CBM32	Binding to galactose, lactose, polygalacturonic acid and LacNAc	137	63	23
		CBM35	Arabinanase; feruloyl esterase D (Fae1;XylD;XynD;CJA_3282); pectate lyase (PelA;CJA_3104)	27	17	10
		CBM61	Modules of approx. 150 residues found appended to GH43, GH53 catalytic domains	3	3	2
		CBM66	Pectate lyase (PecB;Athe_1854;Cbes_1854;Cbes1854)	63	27	13
Total			Families	33	31	28
			ORFs	1756	843	398

^a^RSA (Rice Straw-Adapted) microbial consortia adapted to rice straw from Ref. [44]

^b^EMSD5 microbial consortia adapted to corn stover from reference of [52]

**Fig. 7:**
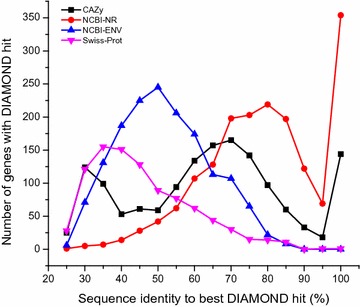
Similarity distribution of putative pectinolytic candidates (*n* = 1756) containing a catalytic domain (CD) or a carbohydrate-binding module (CBM) associated with pectinolytic activity. Sequences were compared to the NCBI-NR (*red* 1756 hits), CAZy (*black* 1464 hits), NCBI-ENV (*blue* 1560 hits), and Swiss-Prot (*pink* 927 hits) databases (best BLAST hit, *E* value ≤1e^−5^); 26 genes contained both a CD and CBM, whereas 1498 and 232 genes contained only a CD or CBM, respectively

To assess the identity of these possible pectinolytic enzymes with known proteins, these amino acid sequences of 1756 putative pectinolytic genes were searched against NCBI non-redundant (NCBI-NR), CAZy, NCBI environmental (NCBI-ENV), and Swiss-Prot databases by DIAMOND [6] (Fig. 7; Additional file 8: Table S6). Firstly, the results based on NCBI-NR showed that the amino acid sequence identity of these 1756 genes ranged from 25 to 100%, with an average of 76.95% (Additional file 8: Table S6). And 23.29% of these sequences were most similar to proteins annotated as “hypothetical/predicted protein” or “proteins of unknown function” in NCBI-NR. Secondly, only 9.83% of these sequences were highly similar (>95% sequence identity) to any CAZy database entry, indicating that most of these sequences had not been previously deposited in CAZy [15]. And 129 sequences were considered as novel with less than 30% identity [31]. Thirdly, only 1.92% of these putative pectinolytic genes are more than 75% identical to sequences deposited in the NCBI-ENV database, demonstrating that these enzymes also have not been observed in previous metagenome projects [15]. Lastly, 145 sequences had less than 30% identity to any known proteins deposited in Swiss-Prot, indicating that their assigned activity has not been verified biochemically. Summarily, the large amount of relatively low identity sequences indicate that the strategy we adopted has great potential in mining novel enzymes from environmental sources.

### Mining for pectinolytic microbes

To explore the phylogenetic origins of these pectinolytic enzymes, we examined the top BLASTX hit organism of each identified enzyme at species level, deciphered the role of individual pectinolytic microbe and their potential synergistic action in the process of pectin degradation. Of the 1756 sequences encoding pectinolytic enzymes, most of their phylogenetic affiliations predicted by BLASTX were consistent with the predicted source organisms of APACMC metagenomic bins (Additional file 7: Table S5). Many of these genes are homologous to those found in the top-10 abundant community members (Fig. 5c), such as *Sphingobacterium* sp. *21*, *Sphingobacterium spiritivorum*, *Thermobacillus composti*, and *Cytophagaceae bacterium SCN 52*-*12*, which further verified that APACMC was successfully target-enriched for pectin degradation.

To provide a systematic overview of pectin degradation by individual member of APACMC, the specific taxonomic assignments of key pectinolytic enzymes, i.e., PGs, PMEs, PNLs, PELs, α-l-rhamnosidases, and arabinofuranosidases, were illustrated in Fig. 6. Clearly, pectinolytic species in APACMC were considerably diverse. The majority of candidate PGs was mainly originated from a variety of *Bacteroidetes* species, which consisted of *Parapedobacter composti*, *Sphingobacterium* sp. *21, Parapedobacter indicus*, *Sphingobacterium thalpophilum*, as well as *Opitutus terrae* and *Verrucomicrobia bacterium IMCC26134* from the *Verrucomicrobia.* It is generally known that the bacterial sources of PELs and PNLs are some specific bacteria such as *Bacillus* sp. and *Pseudomonas* sp. [5]. However, in our study, a broad range of other bacteria were the major producers, including *Parapedobacter composti*, *Sphingobacterium* sp. *21* and *Sphingobacterium thalpophilum*, *Opitutaceae bacterium TSB47*, *Thermobifida fusca TM 51*, *Saccharomonospora glauca*, *Nannocystis exedens*, and *Mesorhizobium* sp. *LC 103*. The taxonomic classifications of genes encoding PMEs and PAEs revealed that they were dominantly from *Sphingobacterium* sp. *21*, *Parapedobacter composti*, *Sphingobacterium thalpophilum*, *Cytophagaceae bacterium SCN 52*-*12* of *Bacteroidetes*, *Pseudoxanthomonas* sp. *J35*, *Cellvibrio mixtus*, *Nannocystis exedens* of *Proteobacteria*, *Saccharomonospora glauca*, and *Ruania albidiflava* of *Actinobacteria* along with *Thermobacillus composti* of *Firmicutes*. The sequences encoding α-l-rhamnosidases and arabinofuranosidases were predominantly affiliated with organisms from *Parapedobacter composti*, *Sphingobacterium* sp. *21* and *Parapedobacter indicus* of *Bacteroidetes*, *Streptomyces caeruleatus* and *Ruania albidiflava* of *Actinobacteria*, and *Thermobacillus composti* of *Firmicutes*. These collective data indicate a synergistic action of multiple members derived from *Bacteroidetes*, *Proteobacteria, Actinobacteria, Firmicutes*, and *Verrucomicrobia* in the degradation of pectin.

Most of the currently available pectinolytic enzymes are reported from filamentous fungal species (e.g., *Aspergillus* sp. and *Penicillium* sp.). However, as the wide functional diversity, broad array of terminal electron acceptors, high ability to degrade lignin [37], as well as more amenable to genetic manipulation, pectinolytic bacteria are likely to play important roles in future biotechnology strategies. Surprisingly, a variety of bacteria were identified as the major producers of various pectinolytic enzymes, such as *Cellvibrio mixtus*, *Cytophagaceae bacterium SCN 52*-*12*, *Nannocystis exedens*, *Opitutaceae bacterium TSB47*, *Parapedobacter composti*, *Parapedobacter indicus*, *Ruania albidiflava*, *Saccharomonospora glauca*, *Sphingobacterium* sp. *21*, *Sphingobacterium thalpophilum*, and *Thermobacillus composti*. Many of these species were initially described to degrade pectin. Strikingly, *Parapedobacter composti*, *Sphingobacterium* sp. *21*, and *Sphingobacterium thalpophilum*, which were identified as the top-10 richest members, each of them harbors a great number of genes encoding various pectinolytic enzymes, indicating that they are well equipped with systematic pectinolytic enzymes. This make them to be promising bacterial sources of pectinolytic enzymes and potential efficient degraders of pectin in the future. Although putative pectinolytic enzymes have been annotated in the genomes of these type-strains [14], so far the information available on the pectinolytic enzymes of these strains is very limited. Our data provide insight into their potentials and highlight their importance in the complex degradation of pectin.

## Conclusions

Novel pectinolytic microbes and enzymes have potential application in numerous industrial processes. Here, we adopted a strategy which combined metagenomics sequencing with enrichment culture technique to rapidly discover efficient pectin degraders and novel pectinolytic enzyme sequences. The immensely diverse pectinolytic microbes and enzymes found in our study will not only shed light on the current understanding of microbial interaction and enzymatic synergism in pectin degradation, but also expand the arsenal of proficient degraders and enzymes for lignocellulosic biofuel production. When combined with high-throughput strategies, such as cell-free protein expression system, droplet-based microfluidics, fluorescence-activated cell sorting (FCAS), and nanostructure-initiator mass spectrometry (NIMS), the efficiency of this strategy for obtaining novel enzymes may meet the ever-growing demand from various industries.

## Methods

### Enrichment of apple pomace-adapted microbial community in compost habitat

The composting materials were composed of apple pomace and fresh cattle manure. Apple pomace was kindly provided by Shaanxi Haisheng fresh fruit juice Co. Ltd., China [46]. The cattle manure was collected from the Northwest A&F University farm located in Yangling, China. The composting experiment was conducted from September 15 to October 29, 2016 according to Sun et al. [41] with slight modifications. Briefly, the cattle manure was mixed with apple pomace to adjust the C/N ratio to 30:1 and the moisture content to around 60% and then the mixture was placed in rectangular foam containers as described in Sun et al. [41]. The piles of compost were turned and sampled daily. Samples were pooled from the top, middle, and bottom of the composting, and then mixed completely. When the pile temperature dropped to ambient temperature at the end of the maturing stage, the composting process was considered as completed. The each sample was split into two parts: one part was stored at 4 °C for subsequent physicochemical analysis and the other was stored at −80 °C for high-throughput sequencing.

### Physicochemical analysis

The pile temperature was monitored every 24 h by inserting a mercury thermometer in the center of the composting material. The moisture content was measured gravimetrically after drying samples at 105 °C for 24 h. The pH values of the samples were tested in water (solid-to-water ratio of 1:10, w/v) with a pH meter [51]. The contents of pectin were determined by modified carbazole method [46].

## 16S rDNA sequencing and phylogenetic classification

The 16S rDNA sequencing was performed at the Frasergen Genoimcs Institute (Wuhan, China) using the Illumina MiSeq platform [33]. The 16S V3-V4 region was amplified using the primers 338F and 806R. After the processing of raw data, the high-quality sequences were subjected to filter singletons, remove chimeras, and cluster into operational taxonomic units (OTUs) at a 97% identity using UPARSE [10]. A representative sequence of each OTU was assigned to a taxonomic level in the SILVA database [35] using the RDP classifier. Microbial diversity and richness measurements were performed using MOTHUR [39]. The microbial diversity was estimated by Shannon and Simpson, and the richness was determined by Chao and Ace estimators.

### Metagenome sequencing, de novo assembly, and Open Reading Frames (ORFs) prediction

Metagenome sequencing, *de novo* assembly, and ORFs prediction were performed by Frasergen Genoimcs Institute (Wuhan, China) according to Qin with slight modifications [34]. Briefly, a library with 400-bp clone insert size was constructed and sequenced on Illumina HiSeq4000 platform. Sequence reads were quality trimmed to an accuracy of 98.0% and duplicate reads were identified and removed prior to assembly. Nearly 89.6 million high-quality reads were generated (16.2 Gb). High-quality short reads of the DNA sample were assembled by the SOAPdenovo assembler [22] with a k-mer length of 39–47. The assembled contigs longer than 500 bp were subject to ORFs prediction using the MetaGene [29] with default parameters. The redundant ORFs were removed by CD-HIT [13] from the non-redundant gene catalog and the abundances were annotated by SOAPaligner [22].

### Taxonomic assignment and functional classification in metagenomic database

Taxonomic annotation of predicated genes was performed by BLASTP against the NCBI-NR database with an E value of 1e^−5^. The APACMC metagenomic dataset was also taxonomically profiled at species level by MetaPhlAn2 [42], MG-RAST [27] and MEGAN 6 [17]. The phylogenetic tree was generated using iTOL software [21]. Functional classification of predicted gene was performed by BLASTP against eggNOG database and by KOBAS 2.0 (a Orthology Based Annotation System) [48] against KEGG database. The “function comparison” module of integrated microbial genomes with microbiome samples (IMG/M) [7] were applied to compare the COG category of APACMC against another four well-known lignocelluloses-degrading microbiomes available on IMG/M, including rain forest compost (IMG Submission ID 5968), switchgrass-adapted compost [2], sao paulo zoo park compost [24], and rice straw-adapted compost [36].

### Carbohydrate-active enzymes (CAZymes): annotation and phylogenetic analysis

Searches for CAZymes were performed as described by Wang and coworkers [44]. Briefly, the amino acid sequences of the predicted ORFs in the APACMC metagenome were annotated by dbCAN, an automated CAZyme signature domain-based annotation method based on family-specific HMMs [49] by MAFFT and HMMER. After identification, these sequences were searched against NCBI non-redundant (NCBI-NR), CAZy database, NCBI environmental database (NCBI-ENV), and Swiss-Prot database by DIAMOND [6] with a cutoff of *E* value <1e^−5^. The phylogenetic distributions in the top ten abundant members possessing CAZymes were visualized via software Circos [20] at the level of phylum, genus, and specie.

### Specific pectin-degrading genes: annotation and phylogenetic analysis

The predicted sequences encoding pectinolytic enzymes were re-annotated and verified using DIAMOND using a sensitive setting [6] against the proteins deposited in NCBI-NR database. The phylogenetic origins of candidate genes were determined by MEGAN 6 [17].

### Sequence data submission

The assembled metagenome datasets were submitted to IMG/M and Metagenomics RAST server (MG-RAST) under the project ID 117466 and mgs566360, respectively.

## Additional files

**Additional file 1: Figure S1.** Red agar test.tif. The effectiveness of enrichment cultures (post- and pre-) by grown on ruthenium red agar plates.

**Additional file 2: Table S1.** Diversity and OUT distributions.xlsx. Diversity and OTU distribution of CM0, Mes5, The15 and Mat30.

**Additional file 3: Table S2.** De novo assembly results.docx. Illumnia reads and de novo assembly results of APACMC metagenome.

**Additional file 4: Figure S2.** The comparison of COG category.tiff. The COG comparison of APAMC with other well-known lignocellulosic metagenomes.

**Additional file 5: Table S3.** Specific COGs.xlsx. Specific COGs envolved in apple pomace deconstruction.

**Additional file 6: Table S4.** Annotation of CAZymes genes.xlsx. Annotation of CAZymes genes by dbCAN.

**Additional file 7: Table S5.** Pectinolytic enzyme genes.xlsx. Catalog of genes encoding pectinolytic enzymes.

**Additional file 8: Table S6.** Distributions of identities.xlsx. Distributions of identities of predicated pectinolytic enzyme sequences.

## Abbreviations

AAs: auxiliary activities
APAC: apple pomace-adapted compost
APACMC: apple pomace-adapted compost microbial community
CAZymes: carbohydrate-active enzymes
CBMs: carbohydrate-binding modules
CEs: carbohydrate esterases
GHs: glycoside hydrolases
COGs: clusters of orthologous groups
GTs: glycosyltransferases
HG: homogalacturonan
PAEs: pectin acetylesterases
PELs: pectate lyases
PGs: polygalacturonases
PLs: polysaccharide lyases
PMEs: pectin methylesterases
PNLs: pectin lyases
MG-RAST: metagenomics RAST
ORFs: open reading frames
OTU: operational taxonomic unit
RGLs: rhamnogalacturonan lyases
RG-I: rhamnogalacturonan I,
RG-II: galacturonans rhamnogalacturonan II
XGA: xylogalacturonan
