# Supplementary figures and images for: Metagenomic mining pectinolytic microbes and enzymes from an apple pomace-adapted compost microbial community
Source: Biotechnol Biofuels. 2017 Aug 22;10:198. doi: 10.1186/s13068-017-0885-y (PMC5568718; doi:10.1186/s13068-017-0885-y)

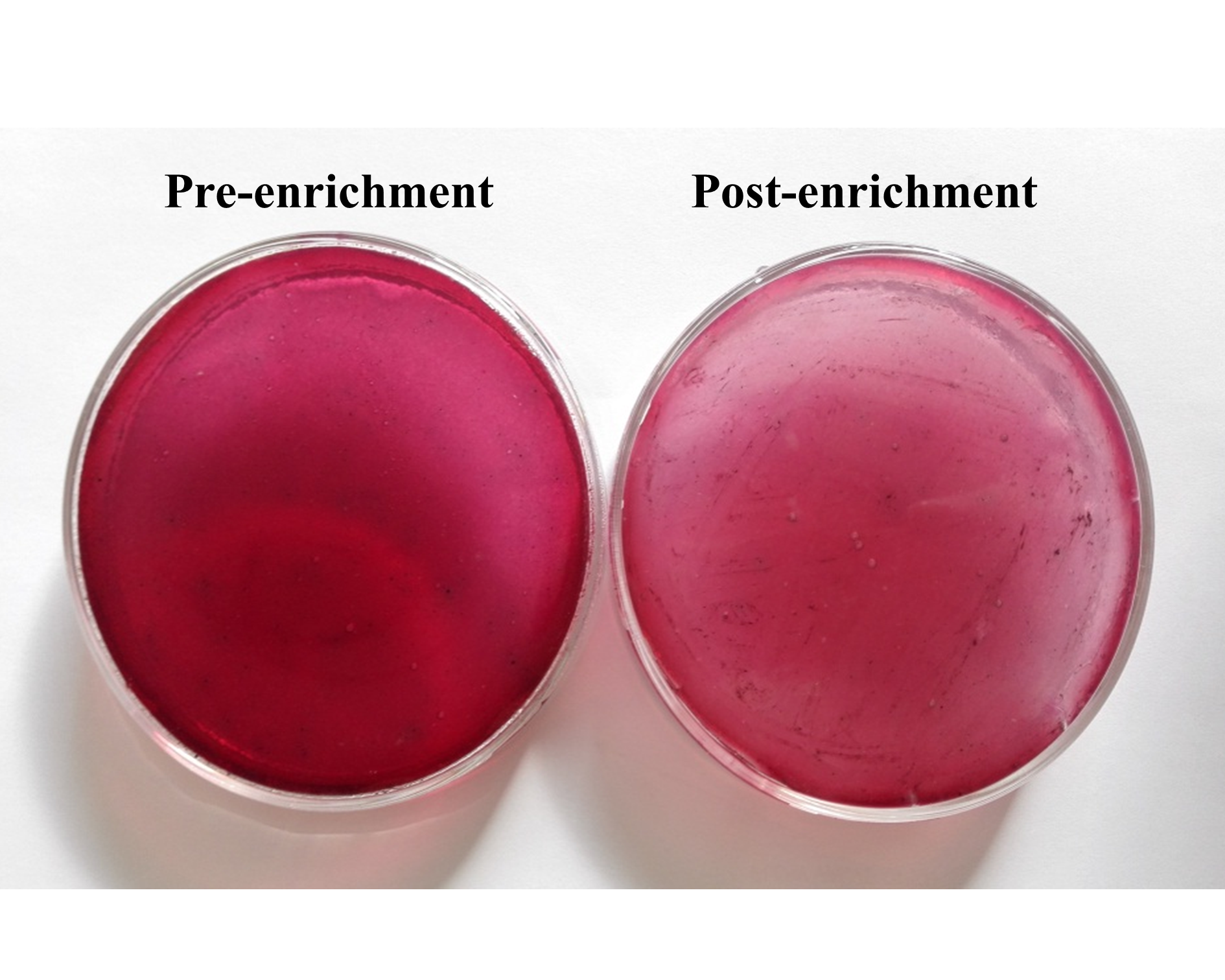

Supplement: Supplementary file 1 — Additional file 1: Figure S1. Red agar test.tif. The effectiveness of enrichment cultures (post- and pre-) by grown on ruthenium red agar plates. [file 13068_2017_885_MOESM1_ESM.tif]

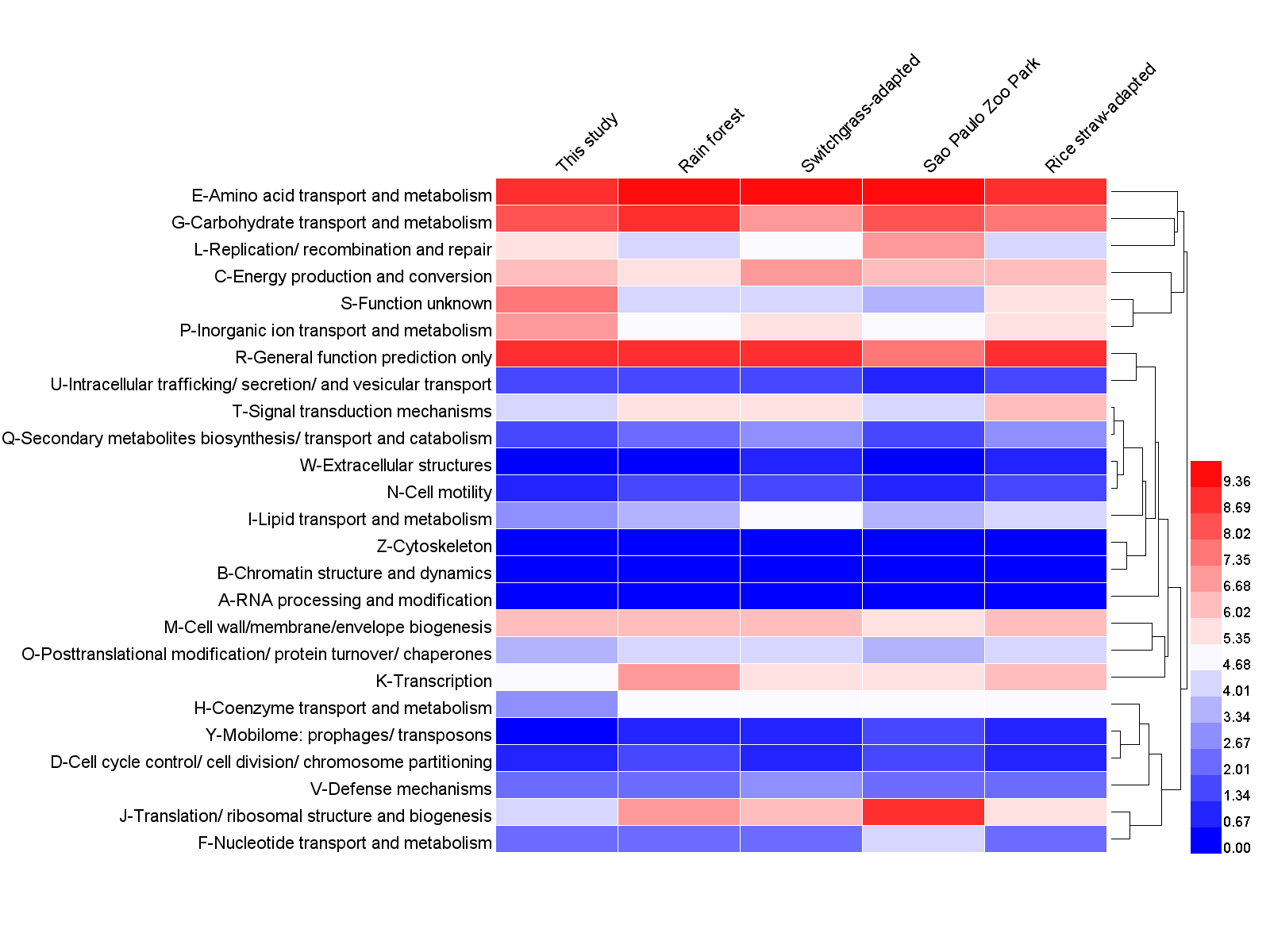

Supplement: Supplementary file 4 — Additional file 4: Figure S2. The comparison of COG category.tiff. The COG comparison of APAMC with other well-known lignocellulosic metagenomes. [file 13068_2017_885_MOESM4_ESM.tiff]
