# Supplementary material for: Metagenomic mining pectinolytic microbes and enzymes from an apple pomace-adapted compost microbial community
Source: Biotechnol Biofuels. 2017 Aug 22;10:198. doi: 10.1186/s13068-017-0885-y (PMC5568718; doi:10.1186/s13068-017-0885-y)
Supplement: Supplementary file 3 — Additional file 3: Table S2. De novo assembly results.docx. Illumnia reads and de novo assembly results of APACMC metagenome. [file 13068_2017_885_MOESM3_ESM.docx]

**Table S2 Illumnia reads and *de novo* assembly results of APACMC metagenome**

| ID | Raw reads | Clean reads | ORFs | Total length  (bp) | Avergage length  (bp) | Max length  (bp) |
| --- | --- | --- | --- | --- | --- | --- |
| APACMC | 91,474,378 | 89,623,103 | 272,516 | 182,127,460 | 668 | 465,819 |
